# Anterior Fontanel Size Among Term Newborns: A Systematic Review and Meta-Analysis

**DOI:** 10.3389/phrs.2021.1604044

**Published:** 2021-05-10

**Authors:** Mohammed Oumer, Ashenafi Tazebew, Mekuriaw Alemayehu

**Affiliations:** ^1^ Department of Human Anatomy, School of Medicine, College of Medicine and Health Sciences, University of Gondar, Gondar, Ethiopia; ^2^ Department of Epidemiology, Institute of Public Health, College of Medicine and Health Sciences, University of Gondar, Gondar, Ethiopia; ^3^ Departments of Pediatrics and Child Health, School of Medicine, College of Medicine and Health Sciences, University of Gondar, Gondar, Ethiopia; ^4^ Department of Environmental and Occupational Health and Safety, Institute of Public Health, College of Medicine and Health Sciences, University of Gondar, Gondar, Ethiopia

**Keywords:** anterior fontanel size, term newborn, mean difference, systematic review, meta-analysis

## Abstract

**Background:** Anterior fontanel is an integral element of an infant craniofacial system. There are six fontanels in the newborn skull, namely anterior, posterior, two mastoid, and two sphenoid fontanels. The anterior fontanel is the largest, prominent, and most important for clinical evaluation. Sex, race, genetics, gestational age, and region are the principal factors that influence anterior fontanel size. There exist inconclusive findings on the size of anterior fontanel in newborns. Therefore, this systematic review and meta-analysis aimed to determine the pooled mean size of anterior fontanel among term newborns and to identify the pooled mean difference of anterior fontanel size between males and females.

**Methods:** PubMed/Medline, Google Scholar, Science Direct, JBI Library, embase, and Cochrane Library databases were systematically searched. All essential data were extracted using a standardized data extraction format. The heterogeneity across studies was assessed using the Cochrane Q test statistic, I^2^ test statistic, and *p*-values. A fixed-effect model and random effect model were used to estimate the pooled mean size of anterior fontanel and the pooled mean difference between male newborns and female newborns, respectively. To deal with heterogeneity, sub-group analysis, meta-regression analysis, and sensitivity analysis were considered. JBI quality appraisal checklist was used to evaluate the quality of studies.

**Results:** In this meta-analysis, 8, 661 newborns were involved in twenty-six studies. Among studies, 13 conducted in Asia, 7 in Africa, 5 in America, and 1 in Europe. The pooled mean size of anterior fontanel was 2.58 cm (95% CI: 2.31, 2.85 cm). The pooled mean size of anterior fontanel for Asia, Africa, America, and Europe region was 2.49, 3.15, 2.35, and 2.01 cm, respectively. A statistically significant mean difference was detected between male and female newborns (D + L pooled MD = 0.15 cm, 95% CI: 0.02, 0.29 cm).

**Conclusion:** The pooled estimate of this review does provide the mean value of the anterior fontanel size in the newborns. There was a statistically significant mean fontanel size difference between male and female newborns. Therefore, male newborns had a significantly larger mean size than female newborns.

## Background

Fontanels are defined as gaps happening when more than two cranial bones are juxtaposed [1–3]. Narrow ridges of fibrous connective tissue, which is called sutures, join the flat bones of the skull [2–5]. Anterior, posterior, two mastoid, and two sphenoid fontanels can be identified in the newborn skull [4–10]. The largest rhomboid anterior fontanel is situated between the two frontal and two parietal bones. This fontanel is the prominent and most important for clinical evaluation [1–3, 5–10]. The mean time of anterior fontanel closure is eighteen months but usually closes by twelve months [5–7, 10]. A place where two or more sutures meet is called the fontanel [10]. The sutures and fontanels in the normal skull, especially anterior fontanel, allow the growth of the brain relative to skull bone growth [1, 2, 9, 10]. Besides, the bones of the skull overlap each other during the labor time for successful delivery; however, the molding process of the skull usually resolved after three to five days of birth [1, 2, 7, 8]. The anterior fontanel is an integral element of an infant craniofacial system [8–10]. The diagnosis of an abnormal fontanel requires an understanding of the wide variation of normal fontanel [7–10]. Knowledge of anterior fontanel size is crucial to identify many disorders. A very small size of the anterior fontanel (or early fontanel closure at birth) can be associated with craniosynostosis and abnormal brain development [8–10]. The large size of the anterior fontanel can be associated with multiple diseases. Of them, skeletal disorders, chromosomal defects and dysmorphogenesis syndromes, endocrine disorders, drug and toxin exposure, fetal hydantoin syndrome, aminopterin induced malformations, congenital infections (rubella and syphilis, for example), and aluminum toxicity [1, 5, 8, 9]. Furthermore, increased intracranial pressure is the most common cause of bulging or delayed closure of the anterior fontanel [8]. A sunken anterior fontanel is the sign of dehydration [1, 8–10]. Anterior fontanel size has been utilized as evidence of altered intracranial pressure, an index of the rate of development, and ossification of the calvarium [11]. It is also an indicator of various medical disorders and abnormal skeletal morphogenesis [1, 2, 7, 11, 12]. The variation in size, shape, and closure time is a key feature of anterior fontanel [8, 11]. Significantly, sex, race, gestational age, genetics, regions, and nutrition are the principal factors that influence anterior fontanel size [10–19]. The developmental anatomy of anterior fontanel is also affected by the rate of brain growth, dural attachment, suture development, and osteogenesis [11]. Incredibly, the difference in the mean size of the anterior fontanel between sexes is inconsistent across different studies. At birth, studies conducted elsewhere reported discrepancies in the mean size of anterior fontanel between sexes. Some of the studies reported that the mean size of the male newborns is significantly larger than the female newborns [7–10, 16, 20, 21]. However, some other reports did not show a significant difference in anterior fontanel size between male and female newborns [11, 13, 15, 22–25].

In different parts of the globe, several studies have been conducted to determine the mean size of the anterior fontanel. However, the studies were inconclusive and there is no concrete evidence established at the global level that pooled the average value of anterior fontanel size. Furthermore, despite there are fragmented studies (presented various local or country-level reference range between sexes) performed across the globe that explore the mean difference of anterior fontanel size between male and female newborns, the findings reported from these studies were controversial and inconclusive. For instance, in most studies, male newborns had significantly larger anterior fontanel size than female newborns. In others, the differences in anterior fontanel size between sexes were non-significant.

Given abnormal fontanel can indicate a serious medical condition [1, 8, 9], it is important to understand the pooled mean size of anterior fontanel and the pooled mean difference of anterior fontanel size between male and female newborns. These findings, pooled average value and pooled mean difference between sexes, provide valuable information to Pediatricians, Anatomists, Neurosurgeons, Neuroradiologists, and other Medical personnel for newborn examination. Therefore, this systematic review and meta-analysis aimed to determine the pooled mean size of anterior fontanel among term newborns and to identify the pooled mean difference of anterior fontanel size between male and female newborns.

## Methods

### Searching Strategies

To avoid duplication, the presence of systematic review and meta-analysis on the topic of interest were checked on different databases (DARE database, Cochrane Library, and JBI Library, for example). PubMed/Medline, Cochrane Library, JBI Library, CINAHL, Google Scholar, Science Direct, Web of Science, and embase databases were systematically searched for relevant studies. Gray literature and other sources were retrieved using Google and Google Scholar searches. Besides, reference lists (bibliographies) of identified studies were checked for the presence of additional studies. Sources including the websites of local libraries were also retrieved. The primary search was conducted in the PubMed database. The search was conducted using the following search strategies (“Anterior fontanelle size” OR “anterior fontanel size” [MeSH Terms] OR “fontanel* size” OR “average size of fontanel*” OR “mean size of fontanel” OR “size of anterior fontanel” [MeSH Terms] OR “anterior fontanelle*” OR “measurement of anterior fontanelle” OR Fontanelles*[MeSH Terms]) AND (“term newborn*” [MeSH Terms] OR “newborn*” OR “term neonate” OR “term infant” OR “term children”). Core search terms and phrases used in different databases were “anterior fontanel size” and “term newborns”.

### Study Inclusion and Exclusion Criteria

The inclusion criteria for this systematic review and meta-analysis were published and unpublished full-text articles in the English language at any time and design. Furthermore, it was included articles referring to healthy term newborns, up to three days of life, with normal birth weight and that reported a mean and standard deviation for anterior fontanel.

It was excluded articles with reference to premature newborns, post-term newborns, low birth weight, macrosomic newborns, with known pathology, multiple pregnancies, and image-based studies.

### Study Outcome and Covariates

The primary outcome of this review was the pooled mean size of anterior fontanel among term newborns. The second outcome was to compare the mean difference of anterior fontanel size between male and female newborns.

### The Methods for Assessing the Size of the Anterior Fontanel

In different nations, there are various methods for assessing the size of the anterior fontanel (traditional method, Area, Oblique diameter, for instance) described as simple clinical methods of measuring anterior fontanel size [2, 7–10]. Many researchers are interested to use the most popular method of Popich and Smith, known as the traditional method [12]. This method is the simplest, practical, and acceptable in clinical settings. To circumvent the problem of the fontanel ended and the suture began, the extent of the anterior fontanel was determined by inserting the index finger in turn into each of the four vertices and a small circular dot was marked with washable ink on the skin immediately distal to the finger. A piece of white paper was firmly pressed over the fontanel so that the four dots were transferred onto the paper [26]. The distance between the anterior and posterior points and between the transversal points was measured and recorded with a fresh ruler. The average of anterior-posterior dimension and transverse dimension was considered as the size of anterior fontanel [10, 12].

### Study Quality Assessment

The Joanna Briggs Institute (JBI) quality appraisal checklist was considered to assess the quality of each study [27]. Two reviewers independently assessed the quality of each study using the tool. Disagreements between reviewers that arise during criticizing the quality of the study were negotiated based on the evidence-based discussions. The JBI critical appraisal checklist for cross-sectional studies was adapted. It contains eight items that are listed from “the criteria for inclusion in the sample clearly defined” to “the appropriateness of the statistical analysis” (Additional file 1). In the end, the study was considered low risk if the study scored fifty and above percent of all quality assessment items of the study design.

### Data Extraction Strategy and Study Selection

After retrieving all studies from the databases, citations were imported into the bibliographic software, Endnote Version 7 Software, to remove the duplicate studies. After the removal of duplicate articles, the remaining studies were screened based on title and abstract for possible inclusion. Full-text articles were deeply reviewed for the entirely to determine the final included article. Two reviewers using a standardized data extraction template extracted all essential data independently. The data abstraction format included primary author, publication year, sample size, country of the study, study design, sex of newborns, mean size of the anterior fontanel, standard deviation, methods, measuring instrument used, male sex (sample size, mean, standard deviation, *p*-value), female sex (sample size, mean, standard deviation), and other parameters.

### Data Synthesis and Presentation

The data analyses were conducted using STATA version 14.1 Statistical Software. The data were extracted in Microsoft Excel and exported into STATA for further analysis.

### Statistical Analysis

#### Meta-Analysis

The heterogeneity across the studies was assessed using the Cochrane Q test statistic (chi-square), I^2^ test statistic, and *p*-values. The heterogeneity was declared as low, moderate, or high when I^2^ test statistics results were 25%, 50%, and 75%, respectively [28]. In the case of estimating the pooled mean size of anterior fontanel, there was no statistically significant heterogeneity among studies (I^2^ = 0.0%, *p*-value = 0.943), therefore, a fixed-effect model was used to estimate the pooled mean size of anterior fontanel [29]. However, in estimating the mean difference between sexes, heterogeneity was detected (I^2^ = 85.5%, *p*-value<0.001). Due to the presence of heterogeneity, a random-effect model (then, sub-group analysis, meta-regression analysis, and sensitivity analysis were considered) was used. A sensitivity analysis was done to observe the influence of a single study on the overall estimation of meta-analysis. Meta-regression analysis was accounted for to identify the source of heterogeneity across the studies. The forest plot and Galbraith plot were used to visualize the presence of heterogeneity among studies. Furthermore, a meta-cumulative analysis was done to display the pattern of effects and the significance of cumulative effect over the publication years. To observe the random variations in time sequence among the studies, a time-trend analysis was undertaken. In all cases, a *p*-value less than or equal to 0.05 was considered statistically significant.

### Assessment of Risk of Bias in Studies

The publication bias was checked by Egger’s regression test and Begg’s test [30, 31]. Statistically significant publication bias was considered if a *p*-value becomes ≤0.05. Egger’s plot was used to visualize the publication bias.

## Results

### Accessed Studies

**FIGURE 1:**
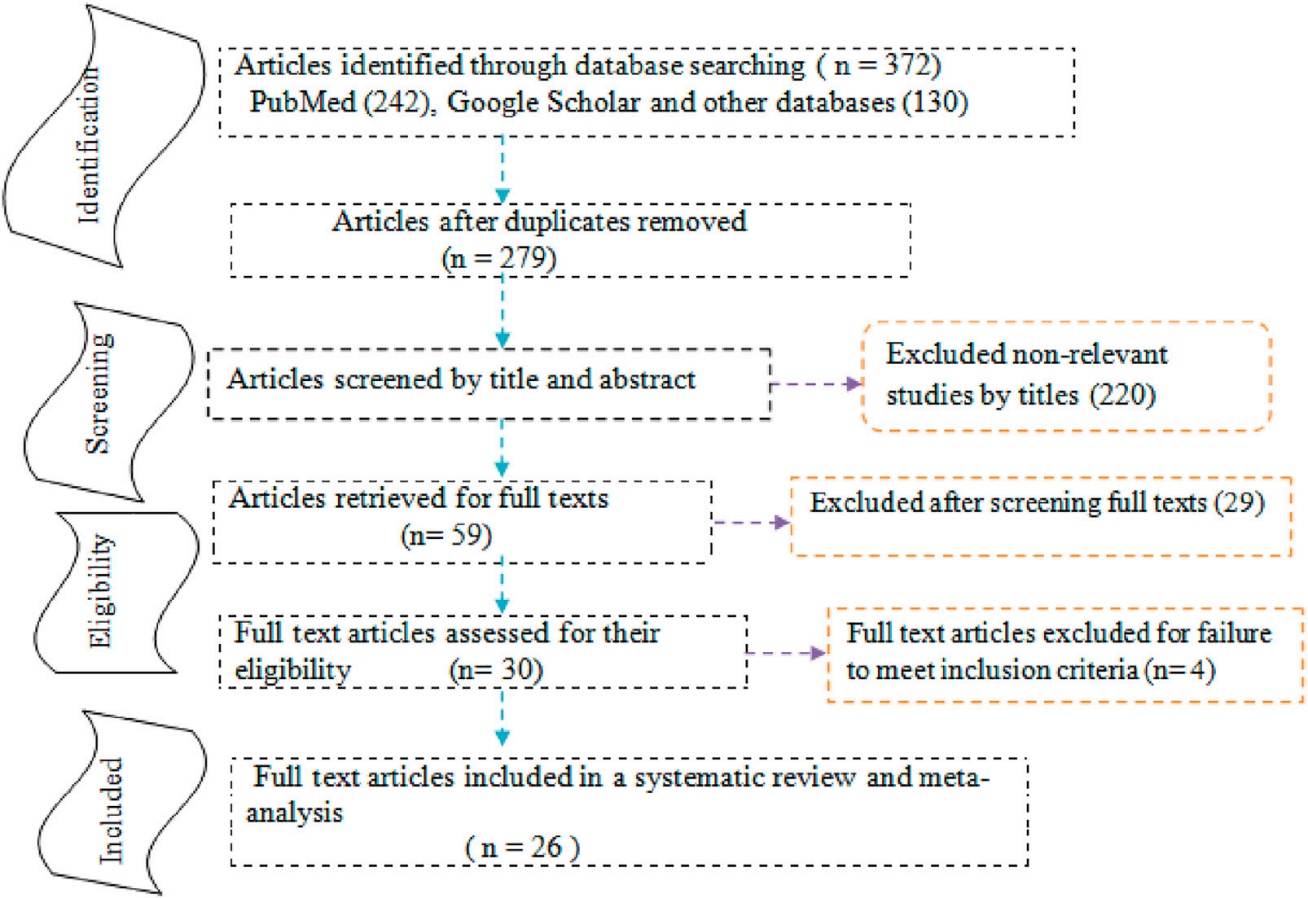
Study selection flow diagram; a figure adapted from the PRISMA group statement in estimating anterior fontanel size.

The reports of this systematic review and meta-analysis were presented based on the preferred reporting items for systematic reviews and meta-analysis (PRISMA) statements [32] (Additional file 2). A total of 372 articles were initially retrieved regarding anterior fontanel size through PubMed, Google Scholar, Science Direct, Cochrane Library, JBI Library, Medline, Embase, and others. Of these, 93 were excluded due to duplicated articles. From the remaining 279 articles, 220 articles were excluded after reviewing its titles and abstracts because titles were found irrelevant for this study. The rest 59 articles were screened for full-texts and 29 were excluded due to the outcome of interest. Therefore, 30 full-text articles were assessed for eligibility based on the pre-determined criteria (four excluded [33–36]). Finally, 26 studies were fulfilled the eligibility criteria and included in the systematic review and meta-analysis (Figure 1).

### Characteristics of the Original Studies

All included studies had used a cross-sectional study design [7–21, 23–26, 37–41]. The studies included a variable number of newborns, ranging from 33 to 2, 215 newborns [21, 26]. The largest study was carried out in Sri Lankan. From all studies, thirteen conducted in Asia [7, 8, 11, 14, 17, 19, 21, 23, 24, 38, 40, 41], seven in Africa [9, 10, 13, 20, 25, 37, 39], five in America [12, 15, 16, 26], and one in Europe [18]. The total size included in this review were 8, 661 newborns. All studies were published in the year between 1972 and 2018. Two articles reported two mean values for two different racial groups (Black American and White American and Hilly and non-hilly Indian). Thus, we included them separately in the analysis [15, 17] (Table 1).

**TABLE 1:** The characteristics of original studies included in meta-analysis for mean size estimation of anterior fontanel, 2020.

First author	Year	Country	Study design	Sample size	Mean	±S.D.	Measuring instrument	Method	Quality status
Uzukwu-edeani [13]	2013	Nigeria	CS	269	2.97	0.71	Steel tape	Trad	Low risk
Faix et al. [15]	1982	America	CS	293	3.08	0.8	Paper tape	Trad	Low risk
Mathur et al. [14]	1993	India	CS	445	3.37	0.61	SC	Trad	Low risk
Chakrabarti et al. [17]	1989	India	CS	110	3.35	1.07	Tape	Trad	Low risk
Chakrabarti et al. [17]	1989	India	CS	130	3.8	1.95	Tape	Trad	Low risk
Tirpude et al. [11]	2016	India	CS	698	4.24	2.21	VC	Trad	Low risk
Mir et al. [20]	1988	Libya	CS	200	2.72	0.63	Steel tape	Trad	Low risk
Faix et al. [15]	1982	America	CS	73	2.67	0.7	Paper tape	Trad	Low risk
Chang et al. [19]	1990	China	CS	79	2.67	5.75	Steel tape	Trad	Low risk
Omotade et al. [25]	1995	Nigeria	CS	337	3.4	0.6	Steel tape	Trad	Low risk
Adeyemo et al. [37]	1991	Nigeria	CS	200	4	1	Tape	Trad	Low risk
Shajari et al. [7]	2011	Iran	CS	400	2.54	1.33	Paper tape	Trad	Low risk
Esmaeili et al. [8]	2015	Iran	CS	208	2.55	1.92	Steel ruler	Trad	Low risk
Perera et al. [21]	2013	SriLanka	CS	2215	2.55	0.92	Plastic tape	Trad	Low risk
Srugo et al. [38]	1987	Israel	CS	303	2.06	0.6	Tape	Trad	Low risk
Popich et al. [12]	1972	America	CS	201	2.1	2	Steel tape	Trad	Low risk
Davies et al. [26]	1976	America	CS	33	220.2	28.6	Steel tape	Area	Low risk
Jackson et al. [16]	2009	Hispanic	CS	170	2.25	7.9	DC	Trad	Low risk
Duc et al. [18]	1986	Switzerland	CS	111	2.01	0.72	Caliper	Oblique	Low risk
G/Meskel [9]	2004	Ethiopia	CS	78	3.35	0.94	Ruler	Trad	Low risk
Oumer et al. [10]	2018	Ethiopia	CS	384	3	0.62	Ruler	Trad	Low risk
Adeyemo et al. [39]	1999	Nigeria	CS	250	3.9	9.65	Ruler	Trad	Low risk
Roy et al. [23]	2018	India	CS	745	2.08	0.45	Ruler	Trad	Low risk
Al-gabban [24]	2008	Iraq	CS	200	2.79	0.71	Tape	Trad	Low risk
Taksande et al. [40]	2015	India	CS	469	2.76	0.55	Tape	Trad	Low risk
Tan KL [41]	1976	China	CS	60	2.05	0.45	Steel tape	Elsasser	Low risk

CS, cross-sectional; Trad, traditional; VC, vernier caliper; DC, digital caliper; SC, sliding caliper; S.D., standard deviation.

Concerning the quality of studies, all included articles were assessed through the JBI quality appraisal criteria. The quality score of the included studies was ranged between fifty percent and ninety percent. Therefore, no studies were included that had considerable risk in the present review (Table 1).

### The Mean Size of the Anterior Fontanel

**FIGURE 2:**
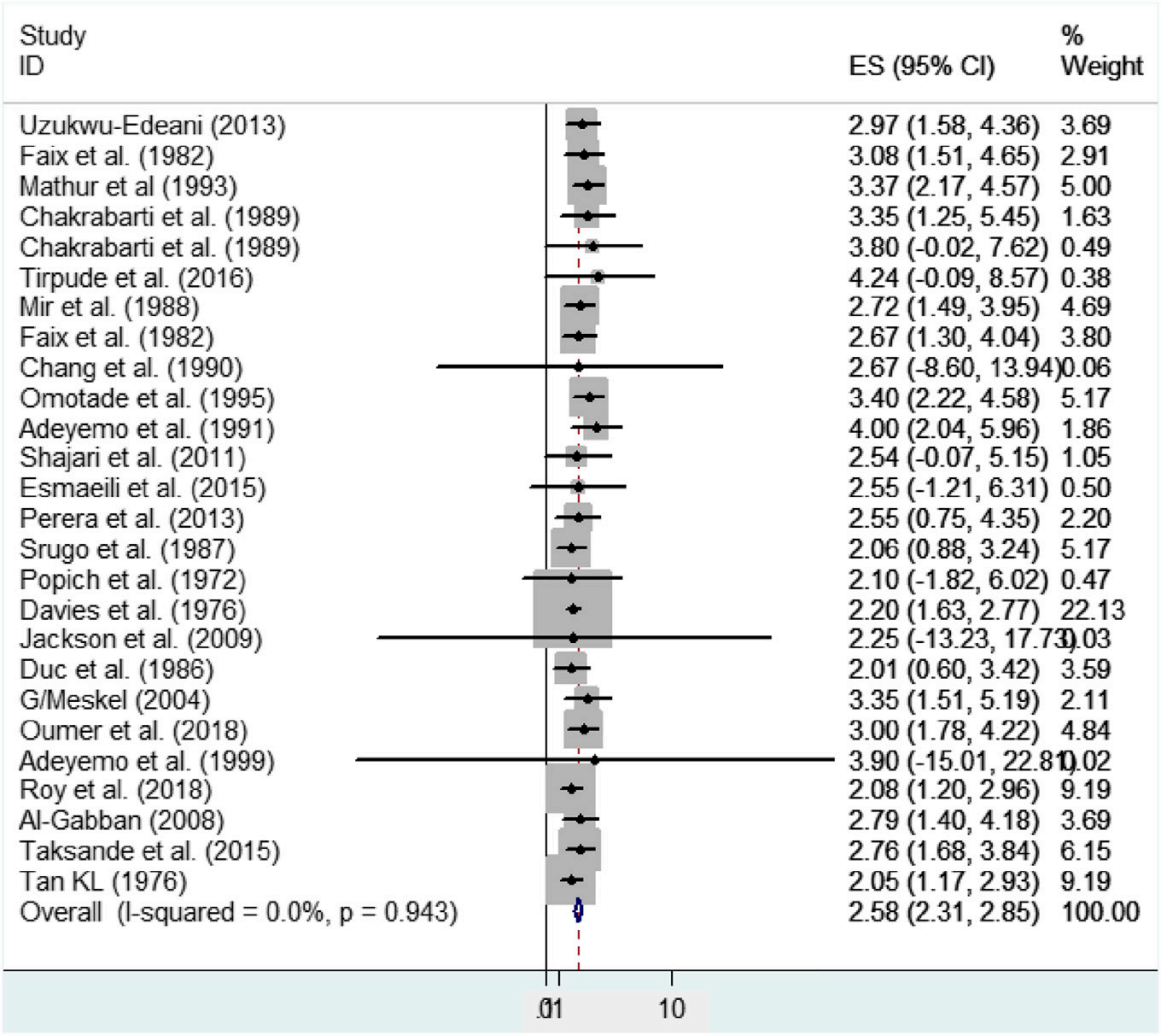
Forest plot showing the pooled mean size of anterior fontanel, 2020.

In this meta-analysis, a significant heterogeneity was not found (effect size attributable to heterogeneity (I^2^) = 0.0%, *p*-value = 0.943, heterogeneity chi-square = 14.9). As a result, a fixed-effect model was applied to calculate the pooled mean size of anterior fontanel. Therefore, the pooled mean size of anterior fontanel was 2.58 cm (95% CI: 2.31, 2.85 cm) (Figure 2).

**FIGURE 3:**
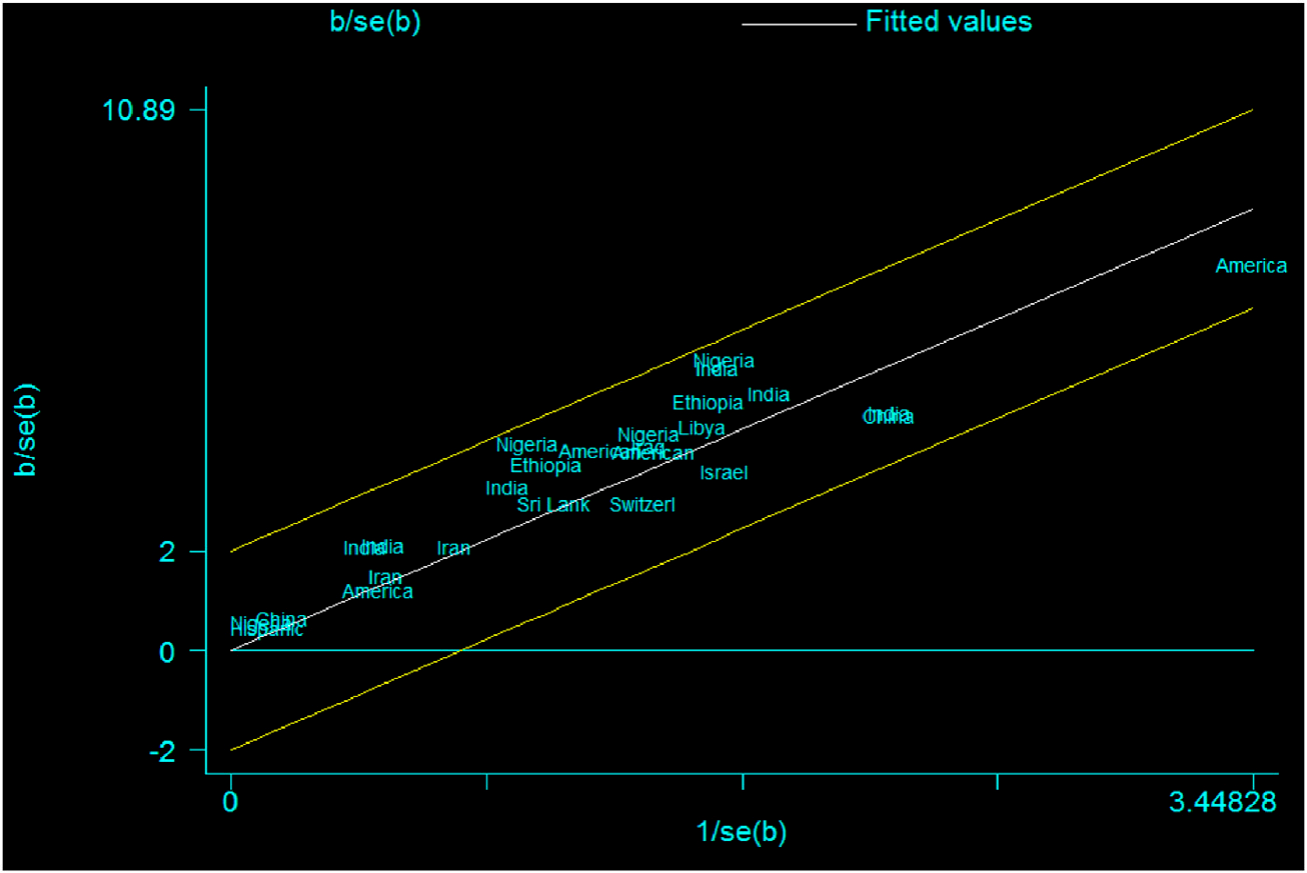
Galbraith plot showing the variability of individual mean size of the anterior fontanel by study country, 2020.

Graphically, the Galbraith plot, outlined all studies based on their country, showed that there is no variability between the studies in the mean size of the anterior fontanel because studies are located within its confidence interval limits (Figure 3).

Based on the study region, the pooled mean size of anterior fontanel for Asia, Africa, United States, and Europe region was 2.49, 3.15, 2.35, and 2.01 cm, respectively (Table 2).

**TABLE 2:** The pooled mean size of anterior fontanel according to the region of the studies, 2020.

S. No	Regions	Mean size of anterior fontanel (95%CI)	I-squared, *p*-value	*p*-value
1	Africa	3.15 (2.58, 3.71)	0.0%, 0.957	<0.001
2	America	2.35 (1.85, 2.84)	0.0%, 0.857	<0.001
3	Asia	2.49 (2.09, 2.89)	0.0%, 0.857	<0.001
4	Europe	2.01 (0.60, 3.42)	–, –	= 0.005
Total	I-V pooled	2.58 (2.31, 2.85)	0.0%, 0.943	<0.001

Based on the study period, the pooled mean size of anterior fontanel for the year between 2011 and 2018 was 2.60 cm (95% CI: 2.09, 3.10; I^2^ = 0.0%, *p*-value = 0.91), for between 2001 and 2010 was 2.99 cm (95% CI: 1.88, 4.10; I^2^ = 0.0%, *p*-value = 0.89), for between 1991 and 2000 was 3.48 cm (95% CI: 2.71, 4.25; I^2^ = 0.0%, *p*-value = 0.96), for between 1981 and 1990 was 2.56 cm (95% CI: 1.99, 3.13; I^2^ = 0.0%, *p*-value = 0.91), and for between 1971 and 1980 was 2.16 cm (95% CI: 1.68, 2.63; I^2^ = 0.0%, *p*-value = 0.96).

**FIGURE 4:**
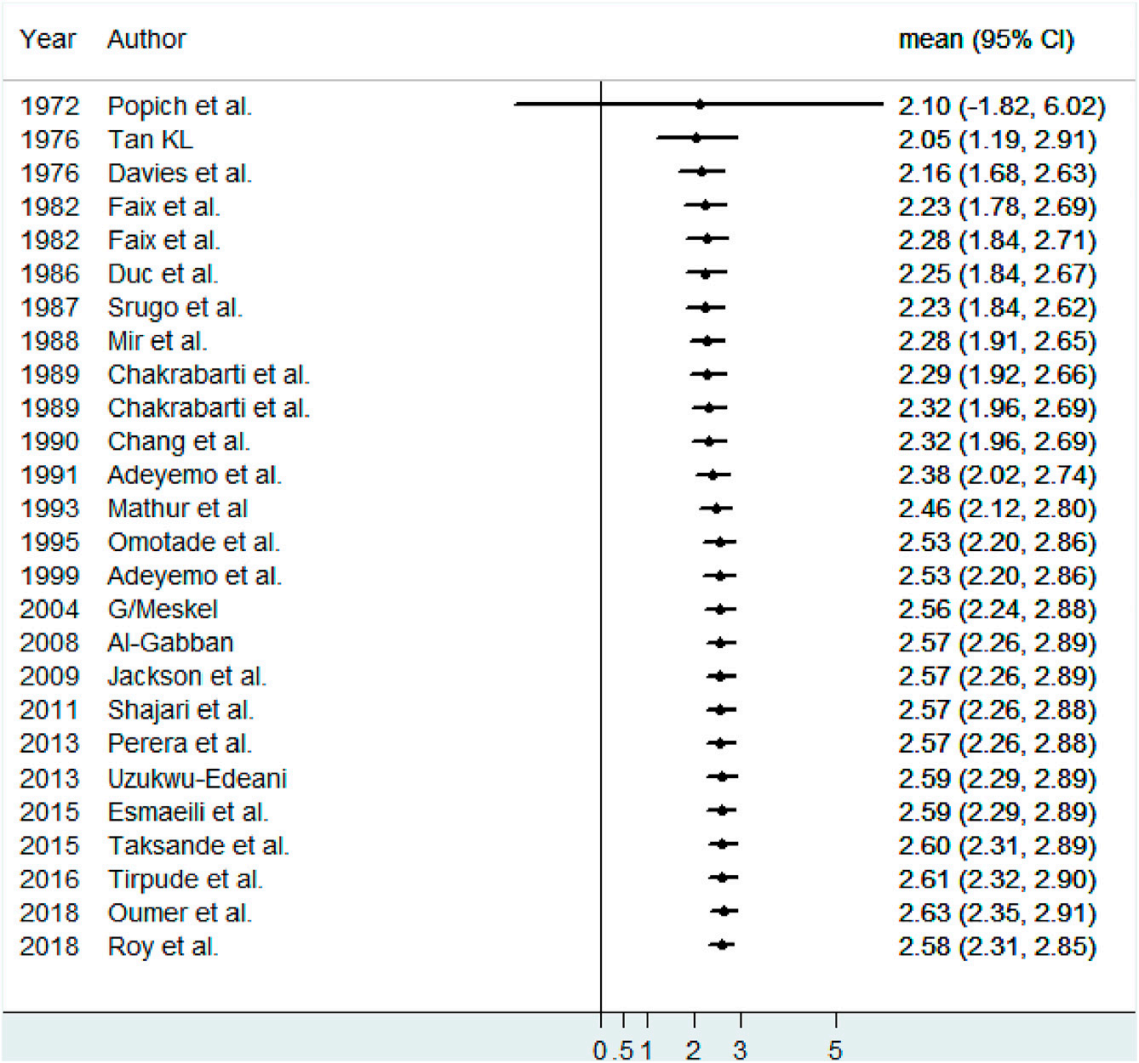
Meta-cumulative analysis showing the cumulative effect of the mean size of anterior fontanel, 2020.

This review described the cumulative effect (using meta-cumulative analysis) of the mean size of anterior fontanel from 1972 (2.10) to 2018 (2.58). Except for the first year, the cumulative effects of all studies were significant (Figure 4).

**FIGURE 5:**
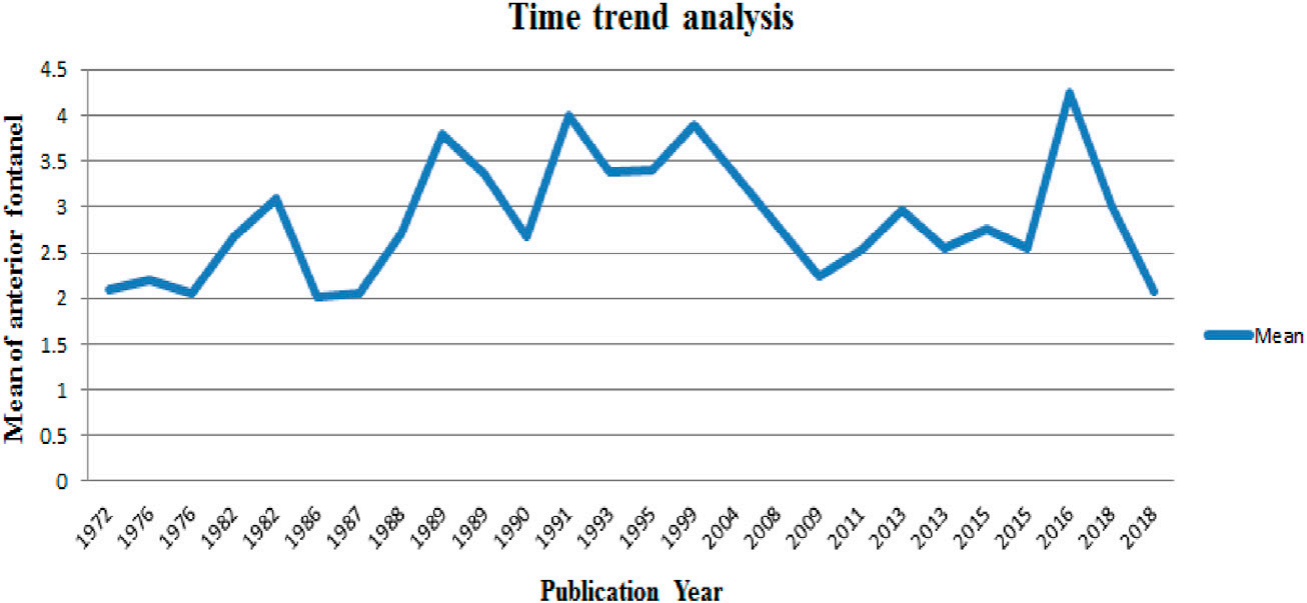
Time trend analysis of the mean value of anterior fontanel in relation to publication year, 2020.

The time-trend analysis showed the relationship between the mean value of anterior fontanel and publication year from 1972 (2.1) to 2018 (2.08) (Figure 5).

### Mean Difference of Anterior Fontanel Size Between Male and Female Newborns

From all studies included in this meta-analysis, twelve original studies [7–11, 13, 18–21, 23, 24] were considered to compare the mean difference of anterior fontanel size between male and female newborns. Seven studies were conducted from Asia [7, 8, 11, 19, 21, 23, 24], four in Africa [9, 10, 13, 20], and one in Europe [18]. The sample size, mean, standard deviation for both males and females were described elsewhere (Table 3).

**TABLE 3:** Descriptive summary of studies included for meta-analysis of mean size difference of anterior fontanel, 2020.

First author	Year	Country	Male			Female			*p*-value	Region
			SS	Mean	S.D.	SS	Mean	S.D.		
Uzukwu-edeani	2013	Nigeria	143	2.97	0.67	126	2.98	0.75	0.89	Africa
Tirpude et al	2016	India	352	4.48	2.26	346	4.02	2.2	–	Asia
Mir et al	1988	Libya	100	2.92	0.51	100	2.51	0.74	0.0001	Africa
Chang et al	1990	China	36	27.2	5	43	26.2	6.5	–	Asia
Shajari et al	2011	Iran	220	2.67	1.32	180	2.37	1.32	0.023	Asia
Esmaeili et al	2015	Iran	110	2.39	0.86	98	2.73	1.02	0.01	Asia
Perera et al	2013	SriLanka	1088	2.57	0.92	1127	2.52	0.92	0.07	Asia
Duc et al	1986	Switzerland	56	19.3	6.6	55	20.9	7.9	–	Europe
G/Meskel	2004	Ethiopia	40	3.53	1	38	3.19	0.85	0.11	Africa
Oumer et al	2018	Ethiopia	206	3.1	0.66	178	2.88	0.57	0.0001	Africa
Roy et al	2018	ndia	547	2.03	0.54	463	2.12	0.55	0.58	Asia
Al-gabban	2008	Iraq	100	2.99	0.73	100	2.58	0.65	0.004	Asia

SS, sample size; S.D., standard deviation.

**FIGURE 6:**
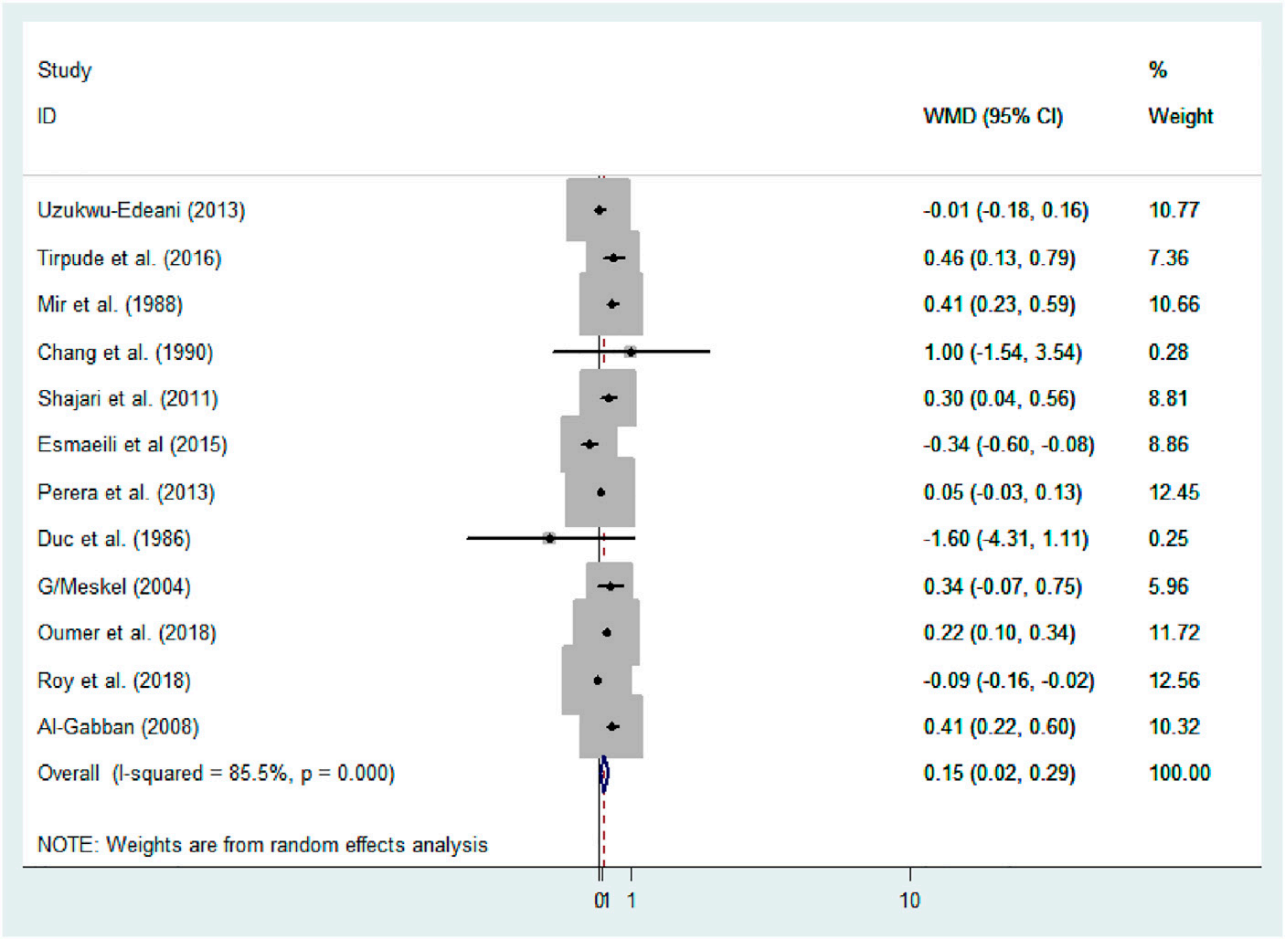
Forest plot showing mean difference of anterior fontanel size, 2020.

Using meta-analysis, significant heterogeneity was found in estimating the mean difference (MD) between male and female newborns (I^2^ = 85.5%, *p*-value<0.001). Consequently, a random-effect model was applied to determine the pooled mean difference of anterior fontanel size (D + L pooled MD: 0.15, 95% CI: 0.02, 0.29). Therefore, male newborns had 0.15 times significantly larger mean fontanel size than female newborns (*p*-value = 0.03) (Figure 6).

The Der Simonian and Laird’s (D + L) pooled prevalence method was considered because the Inverse variance method (I-V) can lead to unreliable estimates. The I-V method assumes that all heterogeneity can be attributed due to the covariates. This assumption may lead to an exaggerated type I error in the presence of residual or unexplained heterogeneity. Unstandardized mean difference (WMD) was used to estimate the effect because all studies were used the same measurement scale, centimeters.

**FIGURE 7:**
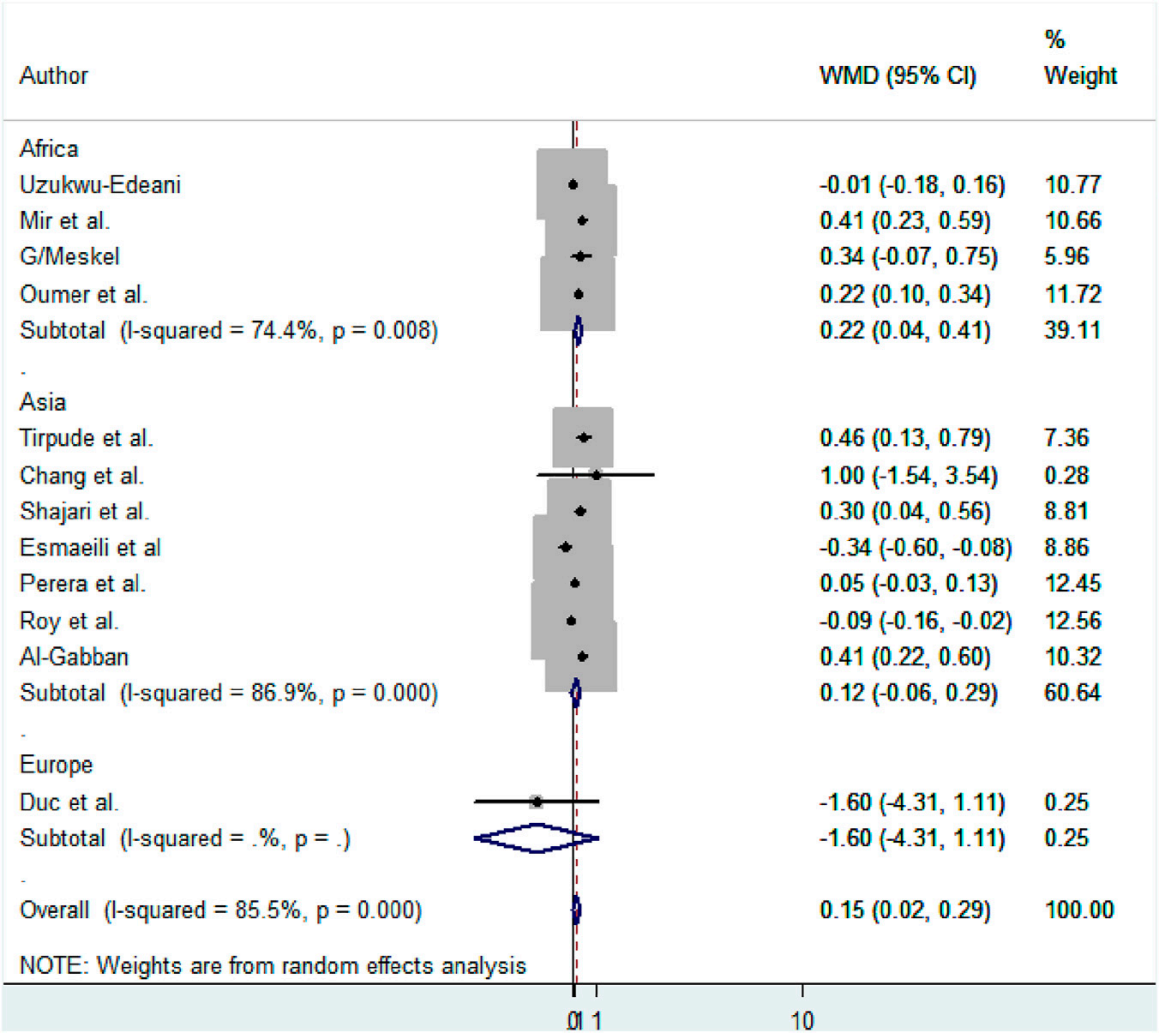
Subgroup analysis by region to show the variability of mean size difference of anterior fontanel among studies, 2020.

**FIGURE 8:**
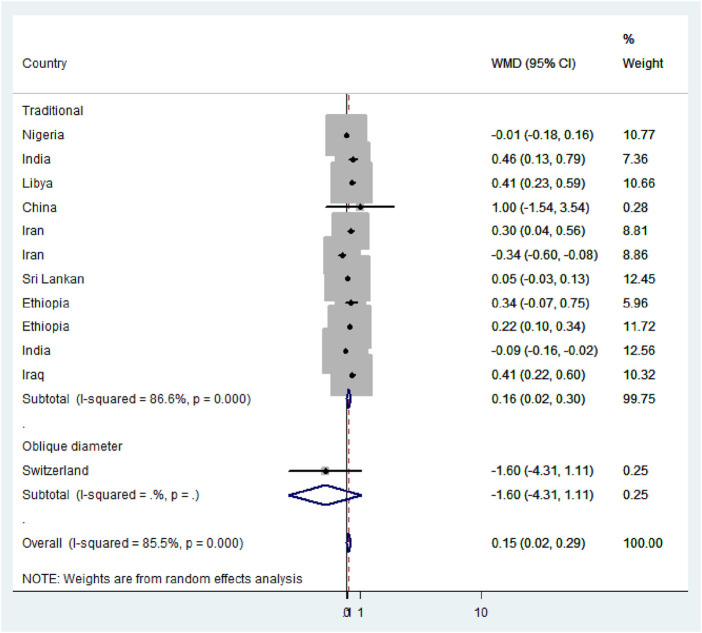
Subgroup analysis by methods, 2020.

**FIGURE 9:**
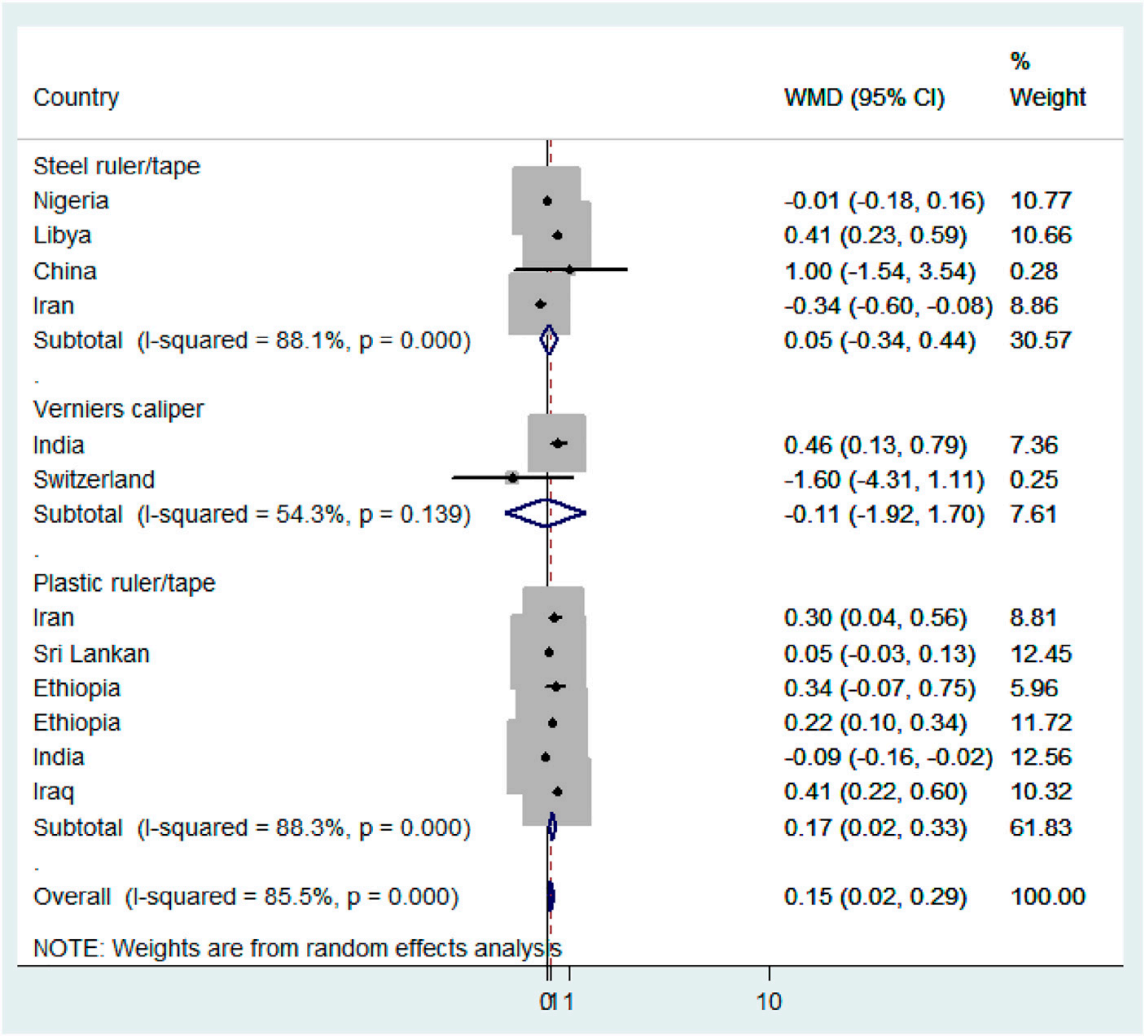
Subgroup analysis by measuring instruments, 2020.

Subgroup analysis based on the region, method, measuring instruments, and the study period was performed to detect the variation in mean difference across the studies. Based on the region, the high mean difference in anterior fontanel size was detected in Africa 0.22 cm (95% CI: 0.04, 0.41) (Figure 7). There was a significant variation in the mean difference of anterior fontanel between study regions (*p*-value<0.001). In the subgroup analysis of methods, there is significant heterogeneity between the traditional and oblique diameter methods (*p*-value<0.001). Because only one study used oblique diameter, it is difficult to predict the magnitude (Figure 8). From measuring instruments, steel ruler (*p*-value<0.001) and plastic ruler or tape (*p*-value<0.001) contribute to the significant variability of mean difference among studies. A statistically significant high mean difference in anterior fontanel size was detected in measuring plastic ruler or tape 0.17 cm (95% CI: 0.02, 0.33) (Figure 9). Based on study period, the mean difference of anterior fontanel size for the year after 2010 was 0.06 cm (95% CI: 0.07, 0.20; I^2^ = 84.9%, *p*-value<0.001), between 2001 and 2010 was 0.40 cm (95% CI: 0.22, 0.57; I^2^ = 0.00, *p*-value = 0.76), and between 1980 and 1990 was 0.32 cm (95% CI: 0.38, 0.03; I^2^ = 13.6, *p*-value = 0.31).

In this meta-regression analysis, sample size (*p*-value = 0.62), study period (*p*-value = 0.77), quality score of studies (*p*-value = 0.65), year of publication (*p*-value = 0.91), study region/country (*p*-value = 0.95), study methods (*p*-value = 0.20), and measuring instruments (*p*-value = 0.26) were analyzed for the source of heterogeneity. None of them was statistically significant.

**FIGURE 10:**
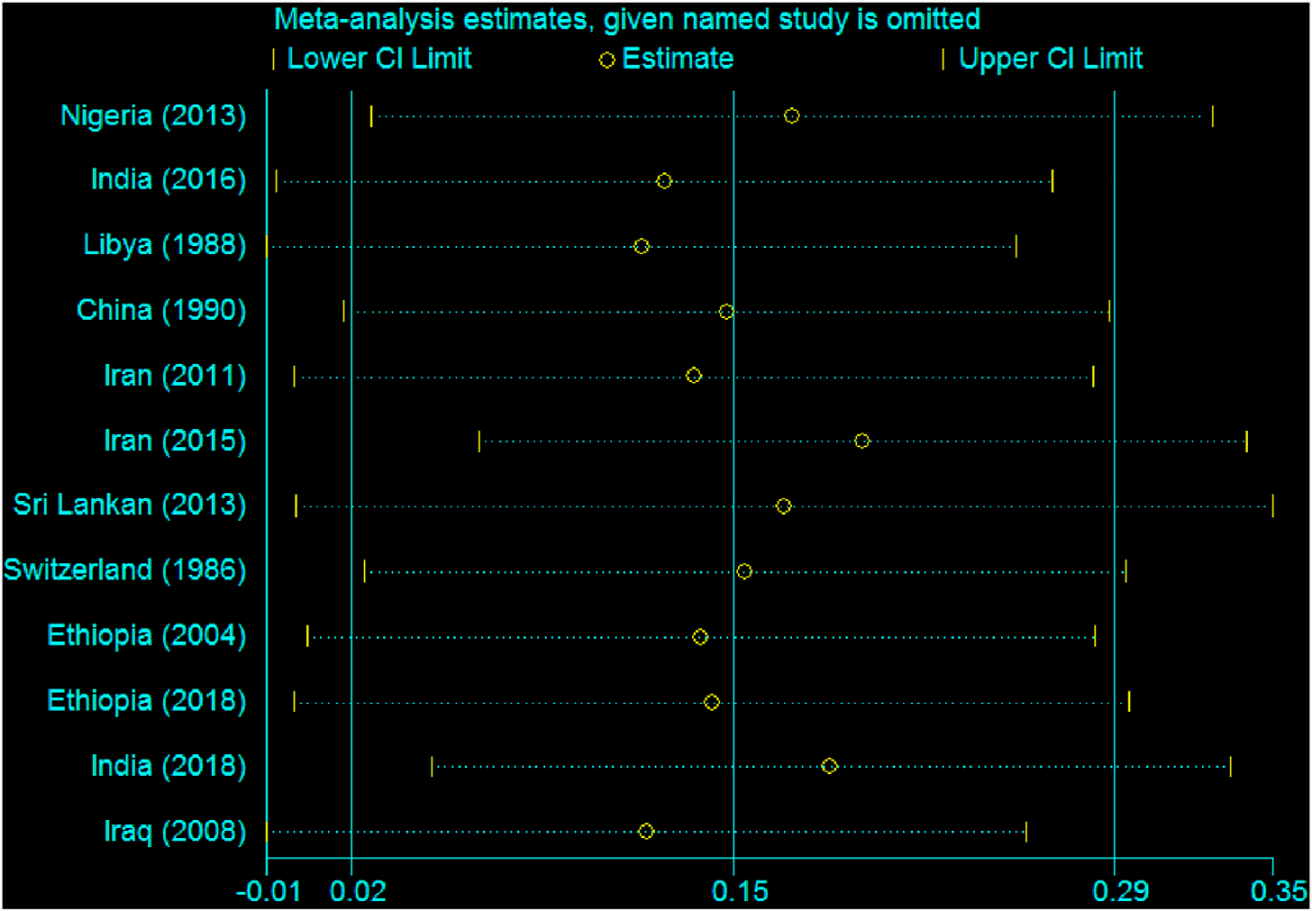
Sensitivity analysis showing the influence of individual studies, 2020.

According to sensitivity analysis, the pooled estimation of this meta-analysis was not influenced by the studies. No individual study influences the overall estimate of the studies (Figure 10).

**FIGURE 11:**
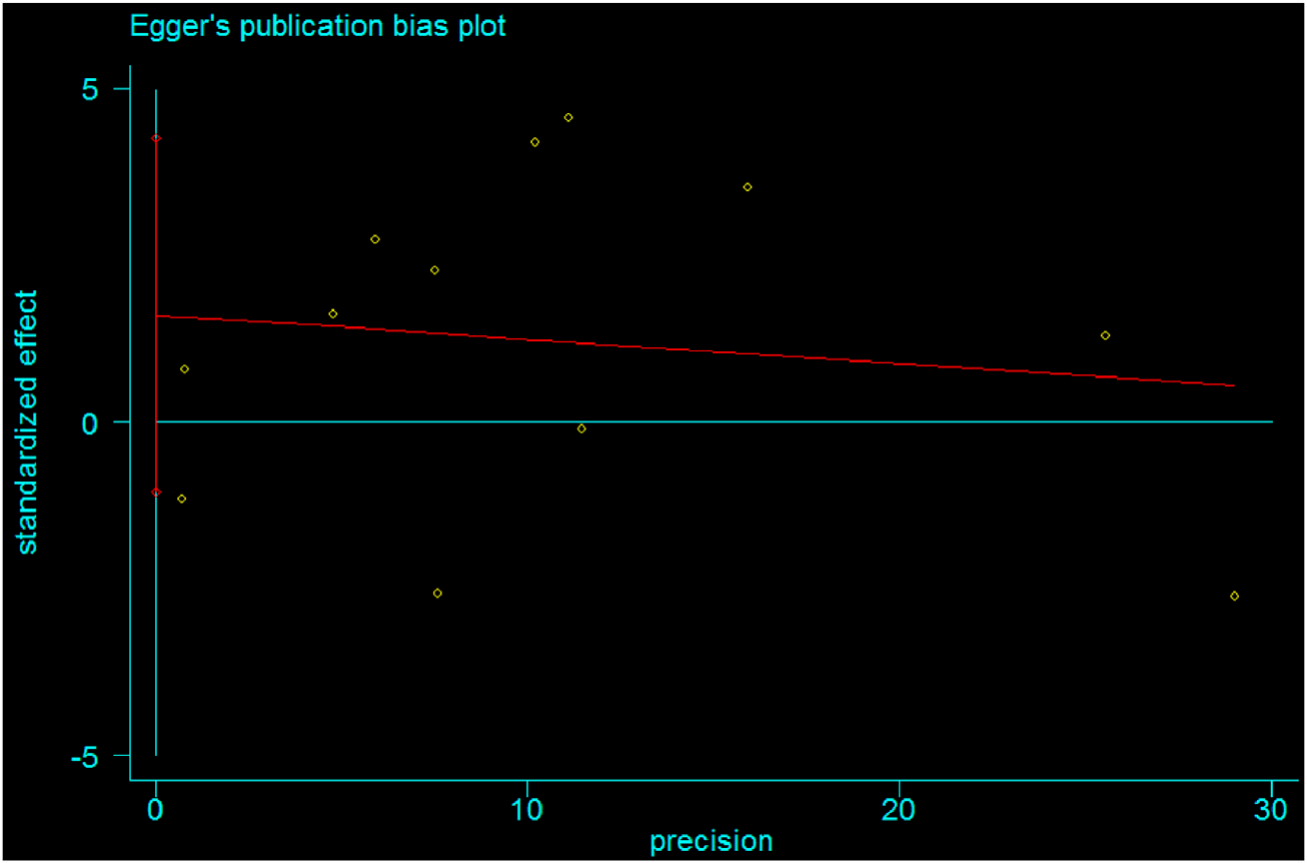
Egger's publication bias plot showing absence of bias among studies, 2020.

Publication bias was also estimated using Egger’s regression tests (B-coefficient of bias = 1.60 (95% CI: 1.1, 4.3); *p*-value = 0.21) and Begg’s test (*p*-value = 0.95). Therefore, there was no statistically significant publication bias in estimating the pooled mean size of anterior fontanel. Besides, Egger’s publication bias plot supports the idea of non-significance (Figure 11).

## Discussion

This systematic review and meta-analysis were employed to determine the pooled mean size of anterior fontanel among term newborns globally based on available studies. Furthermore, it aimed to compare the mean size of the anterior fontanel between male and female newborns. There is a different view among studies regarding the size of anterior fontanel between male and female newborns. The evidence in this review provides the estimates of the pooled mean size of anterior fontanel globally, the overall mean difference between male and female newborns for anterior fontanel size, and the pooled mean size of anterior fontanel for different regions of the world. Interestingly, the findings of this study will have important implications for the clinical examination of the anterior fontanel size among newborns. Thus, it is important to understand the normal variations of anterior fontanel size in different regions and racial groups and the overall pooled reference value globally. Worldwide, everyday physicians carry out a physical examination on thousands of children. Physical examination of anterior fontanel size along with well-child care in neonates is highly recommended medical practices in Pediatrics [7, 8, 10]. It provides important evidence to follow the developmental status of the child and the general state of health. Besides, it can be considered as an index of cranial growth and development during the prenatal and postnatal periods. Any developmental alteration in anterior fontanel growth is an indicator of abnormal growth [7–9, 42].

In the present systematic review and meta-analysis, the pooled mean size of anterior fontanel was 2.58 cm. It can range between 2.31 and 2.85 cm. The heterogeneity across studies was assessed using the Cochrane Q test statistic, I^2^ test statistic, and *p*-values. A fixed-effect model was applied to estimate the pooled mean size of anterior fontanel due to the absence of heterogeneity. Graphically, the Forest and Galbraith’s plot visualized the absence of variability across the studies. In this meta-analysis, the pooled mean size of anterior fontanel for the Asia region was 2.49 cm, for the African region was 3.15 cm, for the America region was 2.35 cm, and for Europe region was 2.01 cm. The larger mean size was detected in the Africa region and a smaller mean size was found in Europe. The number of studies pooled was varied, seven in Africa, one in Europe, thirteen in Asia, and five in America. This review showed that there was no heterogeneity across study regions. Statistically, although there is no variation between the study regions or countries, the difference in the observed value was may be due to the difference in geography, genetics, or race. Besides, sample size and number of studies included in the pooled estimate may have also some influence. For instance, the number of included studies in Europe was only one.

The mean difference between male and female newborns was 0.15 cm. It can range between 0.02 and 0.29 cm. The random-effect model was applied to estimate the overall mean difference between male and female newborns. To deal with heterogeneity, sub-group analysis (based on region, method, measuring instruments, and study period, for example), meta-regression analysis, and sensitivity analysis were considered. The results of these subgroup analyses noted that the mean difference of anterior fontanel is significant among study regions, methods, and measuring instruments. Moreover, meta-regression analysis was performed based on the study region or country, methods, sample size, study period, the quality score of studies, year of publication, and measuring instruments. However, these included covariates in the meta-regression analysis were found not associated with the heterogeneity of the mean difference of anterior fontanel size between sexes. In this review, no study has a special influence over others on the overall estimation of meta-analysis. Essentially, all studies have uniform confidence intervals. A statistically significant mean difference was detected between males and females (MD = 0.15, 95% CI: 0.02, 0.29). The possible explanations for the observed differences in mean value between male and female newborns could be related to the differences in the study region or setting, race, genetics, nutrition, sample size, variation in measuring instruments, variation in methods used, and other methodological differences between the studies. The possible reason may be due to the fact that female newborns mostly had less body size as compared to male newborns (difference in birth weight, head circumference size, gestational age, and other anthropometric measurements).

### Strength and Limitations of the Review

This review provided cumulative evidence in the estimation of anterior fontanel size, which is clinically very important. Besides, it gave a better understanding of anterior fontanel size and its mean difference between male and female newborns. As a limitation, some studies had not clear methodology (under-reported through publication) regarding the normal distribution of values to calculate the mean size of anterior fontanel. We considered only English written articles to meticulously evaluating the quality of the studies. Moreover, the gestational age of newborns (Even if we considered newborns appropriate for gestational age in our criteria, some studies did not explicitly state in the method part for the exclusion of small for gestational age and large for gestational age newborns in their study) and the adequacy of the sample size or variability in sample size may affect the estimated report. Furthermore, in this review, we did not analyze the correlation of anterior fontanel size with other anthropometric measurements at the time of birth (head circumference and birth weight, for instance) due to the inconsistency data related to standard deviations. Even if we performed subgroup analysis and meta-regression analysis for those mentioned covariates to minimize the variability or to identify the effect of covariates, we did not perform these analyses for the race, nutritional status, or antenatal care quantity and quality due to inadequacy of data in individual studies. Once more, this systematic review and meta-analysis considered that differences in strictly following the methods of measurement, such as measuring in the presence of wide sutures and inconsistent use of the same type of measuring instrument throughout the data collection period, may affect the pooled estimate of anterior fontanel size.

### Conclusion

The pooled estimate of this review does provide the mean value of the anterior fontanel size in the newborns. There was a significant pooled mean fontanel size difference between male and female newborns. Thus, male newborns had a significantly larger mean fontanel size than female newborns.

## Data Availability

The data sets used and/or analyzed during the current systematic review and meta-analysis are available from the corresponding author on reasonable request.
